# Is Medical Unity Practical?

**Published:** 1902-09

**Authors:** 


### Is Medical Unity Practical?

MEDICAL unity does not mean unity in medical practice. It is therefore pertinent to inquire, What is medical unity? What is unity in medical practice?

In every department of thought, accuracy is essential to progress and accuracy is the more difficult as similarities increase.

We believe that the medical history of this country would have been written in quite different terms if medical men had never confused medical unity with unity in medical practice. So believing, we ask our readers to take a moment to consider these two thoughts that seem so much alike, but are in fact so unlike.

First, it is to be noted that, as we use the term, medical unity is of the state, is political, and is most closely related to state medicine, to the relations of physicians as a class to the people organised for mutual purpose in that most marvelous conception, the modern state, the commonwealth. That it has to do with the enactment and enforcement of sanitary codes and ordinances, the making and keeping of vital statistics, the reporting of communicable diseases, deaths and causes of death, the giving of expert testimony as to the nature and extent of disease when the same is alleged as the basis of claim for damages, testimony as to testamentary capacity, and testimony as to mental health in cases where insanity is set up as an excuse for crime or to escape the usual penalties for crime, the making of post-mortem examinations in medico-legal cases, serving as medical examiner or coroner, the making of examinations for life insurance, the examinations of applicants for U. S. pension, and the examination for appointment to the medical service of the U.S. army, navy or marine hospital service, and the examination for state license to practise medicine.

For years there has been medical unity in this country in all these various directions, and it is worthy of note that our particular part of the medical profession has not been consulted about the matter, neither has it been brought about in all cases by or with our consent, but has frequently been enacted in spite of our most earnest, emphatic, if unwise, protest. Again, it is to be noted that this and much more that time and space will not permit us to cite, is but the logical consequences of the laws of 1857, 1865, and 1890. That by the operations of these laws and for the above mentioned purposes, the people then ordained that in this state there should be medical unity. We have made sectarians, the laws have made for unity.

Much of the resentment felt by members of our profession against the laws and possibly against the law-making powers, has come from the fact that we have confounded, as many still do, the prerogatives of the law-making authority with the prerogatives of our profession. We have thought, and some still think, that it is our privilege to say who shall practise the art of healing, and what one who so practises shall believe and think. Whereas, the people reserve to themselves the right to declare who shall practise that art and also determine what degree of liberty of opinion, of independence of judgment the members of the craft shall enjoy and exercise.

Again, many have confounded, and some still so confound, the people's efforts at political medical unity with medical uniformity of practice, that the commonwealth was undertaking to dictate to a physician how he should feel, think, and reason, and therefore prescribe or treat in a given case;—the thought of all thoughts the most abhorrent, the most to be opposed, the most subversive of progress, the most unscientific, the most inartistic, the most un-American, the most archaic.

So thinking, good men, simply from mental confusion, from lack of distinguishing between political medical unity and uniformity in medical practice, have drifted into a position more or less out of sympathy with our medical laws, have come to think lightly, harshly, meanly, of the wisdom or expediency of those laws, have ceased from trying to understand or coöperate with such laws, have grown cool, or antagonistic to our county and state medical societies, and have affiliated with other medical bodies having other and less intimate relations to the law-making powers.

This is at one and the same time true, unnecessary and unfortunate. The commonwealth never has, and, while divinely guided, never will endorse this, that or the other theory of therapeutics; never has and never will adopt allopathy, homeopathy or eclecticism, radiography, organotherapy, osteopathy, or Eddyism; but, on the contrary, always has and under an All-wise Providence ever will maintain the right of educated physicians to experiment in therapeutics so long as in medicine there shall be a vestige of the art of healing.

Meanwhile, there seems to be no reason why medical men need to continue to confound things quite easily recognised by their dissimilarities, their individualities; but there is every reason why we should set political medical unity in its place and, remembering its source, with due homage and with sympathetic loyal support, at the same time that we assert and claim as our inalienable and inviolable and imperishable right, that to be free, to be progressive, to be efficient, medical men are and ever must remain the judges of the truths of medicine.

